# Obstetrics

**Published:** 1890-07

**Authors:** Wm. Steinrauf

**Affiliations:** St. Charles, Mo.


### OBSTETRICS.

Wm. Steinrauf, M. D., St. Charles, Mo.

When I read reports of cases of confinement in some homœopathic journals, and the treatment given by the attending physician, I very often feel as if I were reading after an allopathic practitioner. We are there told to have everything done antiseptically ; to use Carbolic acid, Listerine, and the like, quite freely in our examinations and explorations. Then, if after a few days the woman should contract a chill, fever, headache, stoppage of the lochia, etc., we are instructed to wash out the womb with the above-mentioned chemicals, to prescribe Aconite in liberal doses, Bromide of Potassium, Quinine, and light cathartics. Now, if this is Homœopathy, I am willing to confess I don't know what Homœopathy is. It is simply allopathy under a homœopathic cover.

But, says one, I do not think it is right to make warfare against physicians practicing this way. Let every one treat his patients as he sees fit, and his victims give him permission. But when these things are advocated by professed homœopathic physicians, and published in professed homœopathic journals, I think it is no more than right that things should be called by their right names. Surely, no one in his right mind can call such treatment homœopathic !

I think one of the reasons why doctors practice this way is, because they have never thoroughly comprehended the great truths promulgated by Hahnemann. They despise crude allopathy, and have fallen into the track of the eclectics. They go by the name of homœopath, but they have no faith in the dynamized drug; they think disease a material thing, and base their prescriptions on the makeshifts of pathology. Instead of reading and studying the *Organon*, the greatest medical work ever written by human hand, they read old-school journals and practice diluted allopathy.

Where women have had such homœopathic treatment as their most urgent symptoms called for during gestation, and where a few doses of the indicated remedy were taken during labor, they have never had the least trouble in getting up sound and well in from five to eleven days after child-birth. But there has very often been serious trouble when they have been physicked before and after labor, and Chloral and Morphine given to deaden pain in their hour of distress. These and other similar measures are not alone not called for, but absolutely pernicious. And an avowed follower of Hahnemann to sanction the like!

March 2d, 1890, I was called to see Mrs. L., who had been confined by a midwife six days previously. She had been liberally dosed with Senna and Salts twenty-four hours after labor, and when I saw her she had had a severe chill, followed by a very high fever; temperature 105°, and a pulse of 140. Abdomen was very much bloated, the lochia had entirely ceased; she was vomiting, and quite delirious. Treatment, Nux-vom.$^{cm}$, one dose, to counteract the effects of the previous drugging. When I saw her again the next day, the temperature was 103°, and the pulse 110, the abdomen was less tender, vomiting had ceased, and the delirium gone.

The secretion of the mammæ, that had almost ceased, was again re-established; the urine, that had been very scant heretofore, was more profuse, there was less pain when passing it, and the bowels had moved once, and looked more natural. Everything looked more favorable.

I did not think it wise to make a change in the treatment, so I gave another dose of Nux-vom.$^{cm}$ and Sac-lac. for another twenty-four hours. At the end of this period the pulse was 100, and the temperature 101° ; all other symptoms proportionately better. Two days after the temperature rose to 104°, and again the pulse to 130. Where was the trouble? On examination it was found that there was a very severe diphtheritic exudation covering the right tonsil and pharynx. This accounted for the rise in temperature, as the lady had diphtheria, suppressed by allopathy. I became somewhat alarmed at her condition. What shall we do? Give her Iron, Quinine, gargle her throat, paint it with iodine, and treat the name "diphtheria"? No. We shall treat her symptoms according to the unerring law of Homœopathy. Lycopodium$^{cm}$, one dose, and Sac-lac. Two days after taking this one dose of Lycopodium, the temperature was 99°, the pulse 85, and the membrane had disappeared. I heard from her three days later that she was up and well.

Could we expect a better or quicker result by any known method of cure outside of Homœopathy? Could eclecticism do as well?

